# Which one is the best in treating deep venous thrombosis —— percutaneous mechanical thrombectomy, catheter-directed thrombolysis or combination of them?

**DOI:** 10.1186/s13019-024-02908-3

**Published:** 2024-07-05

**Authors:** Hao Zhang, Xiao-ye Li, Jia-si Li, Shi-bo Xia, Chao Song, Qing-sheng Lu, Wei Zhao, Lei Zhang

**Affiliations:** 1 Department of Vascular Surgery, Changhai Hospital, Navy (Second) Military Medical University, Shanghai, 200433 China; 2 Department of Neurology, Changhai Hospital, Navy (Second) Military Medical University, Shanghai, China; 3 Department of General Surgery, The Fifth People’s Hospital of Chengdu, Chengdu, Sichuan Province China

**Keywords:** Percutaneous mechanical thrombectomy, Catheter-directed thrombolysis, Deep venous thrombosis

## Abstract

**Objective:**

To compare the treatment outcomes among percutaneous mechanical thrombectomy (PMT) with AngioJet, Catheter-directed thrombolysis (CDT), and a combination of both.

**Methods:**

One hundred forty nine patients with acute or sub-acute iliac-femoral vein thrombosis accepting CDT and/or PMT were divided into three groups respectively: PMT group, CDT group, PMT + CDT group (PMT followed by CDT). The severity of thrombosis was evaluated by venographic scoring system. Technical success was defined as restored patent deep venous blood flow after CDT and/or PMT. Clinical follow-up were assessed by ultrasound or venography imaging. The primary endpoints were recurrence of DVT, and severity level of post-thrombotic syndrome (PTS) during the follow-up.

**Results:**

Technical success and immediate clinical improvements were achieved on all patients. The proportion of sub-acute DVT and the venographic scoring in PMT + CDT group were significantly higher than that in CDT group and PMT group (proportion of sub-acute DVT: *p* = 0.032 and *p* = 0.005, respectively; venographic scoring: *p* < 0.001, respectively). The proportion of May-Thurner Syndrome was lower in PMT group than that in CDT and PMT + CDT group (*p* = 0.026 and *p* = 0.005, respectively). The proportion of DVT recurrence/stent thrombosis was significantly higher in CDT group than that in PMT + CDT group (*p* = 0.04). The severity of PTS was the highest in CDT group ( χ^*2*^ = 14.459, *p* = 0.006) compared to PMT group (*p *= 0.029) and PMT + CDT group (*p* = 0.006).

**Conclusion:**

Patients with sub-acute DVT, high SVS scoring and combined May-Thurner Syndrome were recommended to take PMT + CDT treatment and might have lower rate of DVT recurrence/stent thrombosis and severe PTS. Our study provided evidence detailing of PMT + CDT therapy.

**Supplementary Information:**

The online version contains supplementary material available at 10.1186/s13019-024-02908-3.

## What this paper adds

Indication and effect of percutaneous mechanical thrombectomy (PMT) + catheter-directed thrombolysis (CDT) in treating DVT is not clear. This study compared PMT only, CDT only and combination of PMT + CDT treatment. The results showed patients with sub-acute DVT, high society of vascular surgery (SVS) scoring and combined May-Thurner Syndrome may benefit from PMT + CDT treatment and might have lower rate of DVT recurrence/stent thrombosis and severity level of PTS.

## Introduction

Deep venous thrombosis (DVT) has a high rate of morbidity and mortality [1]. Besides early complications like pulmonary embolism (PE), the late complications, such as recurrent thrombosis and post-thrombotic syndrome (PTS) can also develop into lifelong diseases [2, 3]. Researchers have increasingly focused on iliofemoral DVT to assess the best treatment strategy for these complications. Anticoagulation therapy is an effective treatment against progression of thrombosis and pulmonary embolism [4]. However, most of these patients’ venous have residual thrombus [5], and there is high incidence of recurrent thrombosis (2%-10%) [6] and PTS (20–50%) after anticoagulation treatment [7].

With the development of new endovascular therapy for iliofemoral DVT, percutaneous mechanical thrombectomy (PMT) like AngioJet (Boston Scientific) and Catheter-directed thrombolysis (CDT) have been widely used in iliofemoral DVT treatment, which can rapidly reduce thrombus burden, potentially preserve venous function and reduce the risk of PTS. However, which treatment is better is still controversial in different studies [8–10]. Only few studies have focused on the comparison of PMT, CDT, and PMT combined with CDT [10]. In this study, we aim to compare the clinical efficacy and outcomes of PMT, CDT, combination of PMT and CDT in iliofemoral DVT patients retrospectively in a center.

## Materials and methods

### Study participants

This is a retrospective investigation. From October 2015 to October 2018, patients with acute (≤ 14d) or sub-acute (> 14d to ≤ 28d) iliofemoral DVT underwent CDT and/or PMT were enrolled in this study in Changhai Hospital. All enrolled patients had symptoms of swelling or pain and were confirmed by ultrasound or venography [11]. The patients were divided into three groups retrospectively: PMT group (underwent PMT only), CDT group (underwent CDT only), PMT + CDT group (underwent PMT first and then CDT). Exclusion criteria included: recurrent ipsilateral DVT, isolated DVT below the knee, bilateral DVT, contraindication of thrombolytic or anticoagulation drugs, and moderate to severe anemia. The study was performed according to the requirement of our Institutional Ethics Committee (Shanghai Changhai Hospital Ethics Committee) (supplement 1). Each enrolled participant agreed to be included in the study and signed the consent form. Patient’s comorbidities and risk factors were collected (Table 1).

**Table 1:** Patient’s comorbidities and risk factors

Comorbidities and risk factors	CDT (*n* = 65)	PMT (*n* = 49)	PMT + CDT (*n* = 35)	*χ*^*2*^/*F*	*P*
Hypertension	26(40.0%)	15(30.6%)	11(31.4%)	1.326	0.515
Cerebral infarction	7(10.8%)	5(10.2%)	3(8.6%)	0.123	0.940
Diabates	3(4.6%)	2(4.1%)	2(5.7%)	0.123	0.940
PAD	2(3.1%)	1(2.0%)	1(2.9%)	0.122	0.941
Coronary artery disease	4(6.2%)	2(4.1%)	1(2.9%)	0.614	0.736
Surgery or trauma	11(16.9%)	7(14.3%)	3(8.6%)	1.313	0.519
Smoking	5(7.7%)	5(10.2%)	5(14.3%)	1.094	0.579
COPD	0	0	1(2.9%)	3.279	0.194
Immobilization	10(15.4%)	13(26.5%)	6(17.1%)	2.371	0.306
Previous VTE^a^	2(3.1%)	2(4.1%)	2(5.7%)	0.410	0.815
Malignancy	4(6.2%)	3(6.1%)	1(2.9%)	0.568	0.753

Continuous data are presented as means ± standard deviation; Categorical data are given as counts (percentage)

*CDT* catheter-directed thrombolysis

*PMT* percutaneous mechanical thrombectomy

*PAD* peripheral arterial disease

*COPD* chronic obstructive pulmonary disease

*VTE* venous thromboembolism, including deep venous thrombosis (DVT) and pulmonary embolism (PE)

^a^One patient in CDT group and one patient in PMT + CDT group had the history of contralateral DVT. The other four patients in the three groups had the history of PE

### Treatment

All patients received warfarin, new oral anticoagulant (Rivaroxaban, 15 mg, twice daily) or low-molecular-weight heparin (enoxaparin, 150 U/kg per day) on the day of diagnosis according to local routines based on international guidelines [12]. Interventional therapy was performed immediately after venography from the ipsilateral dorsal foot vein. Retrievable inferior vena cava filter (RIVCF, Lifetech Scientific, Shenzhen, China) were placed via the contralateral femoral puncture if the patients with acute, massive pulmonary embolism (PE) and floating thrombus or were considered to be at risk for further PE during the procedure according to our national guideline [13]. The ipsilateral popliteal vein was then accessed using a 6-F Introducer (Terumo Medical Corp, Tokyo, Japan) under ultrasound guidance (SonoSite 180 plus; SonoSite, Bothel, WA, USA) for further treatment.

In the PMT group, the AngioJet system (35 mm × 60 mm, Boston Scientific, USA) was routinely used as previously described [14–17]. After initial antegrade venography via an introducer sheath, the 8F AngioJet catheter was advanced along a 260-cm guide wire and through the thrombosis segment. First, a power pulse lytic model was used with 250 000 units of urokinase (Tianpu Pharmaceutical Biochemical Medicine Co, Ltd, Guangzhou, China) in 50 mL saline solution. 10 min were allowed for the pharmacological thrombolytic effect and the AngioJet catheter was placed in its standard rheolyticthrombectomy mode afterwards. This procedure was repeated if significant residual thrombus remained on the subsequent venography. A maximum 500 ml of suction fluid was used.

In CDT group, multiple-side-hole infusion catheter (Angiodynamics, Queensbury, NY, USA) with an infusion length of 40 to 50 cm was deployed into the thrombotic segment and venography was taken to make sure the location was correct. 250 000 units of urokinase were injected immediately, and then (20,000–30000 U/h) was continuously injected for 24–48 h until the sign of the improvement of symptoms. The activated partial thromboplastin time (APTT) and fibrinogen level were measured every 12 h to adjust the heparin and urokinase dose. The APTT should be 2–3 times higher than the normal value.

In PMT + CDT group, AngioJet thrombectomy was followed by the introduction of a multiple-side-hole infusion catheter into the thrombotic segment and urokinase was then injected (the dose adjustment was the same as CDT group). In this group, patients accepted both pharmacological thrombolytic and standard rheolytic thrombectomy first, and waited for 30-45 min. If the venography showed the residual thrombosis greater than 50% and the time of PMT was over 10 min, CDT would be used increased.

In the three groups, percutaneous transluminal angioplasty (PTA) needed to be performed before stent placement if after CDT and PMT showed that the residual iliac-femoral vein had severe stenosis (May-Thurner Syndrome instead of residual thrombus) > 75%. In CDT group and PMT + CDT group, PTA and stent placement as described above were performed in the secondary operation after CDT if necessary. Warfarin or Rivaroxaban were prescribed for at least 6 months, and the international normalized ratio (INR) was maintained between 2.0–3.0 for Warfarin [12]. All the patients used intermediate-pressure compression stockings (class II, 23–30 mm Hg) as a standard adjunct treatment. Patient demographics, DVT characteristics, individual procedural details and complications were all collected and reviewed for each patient. In this study, the patients should be taken venography to detect RIVCF trapped embolus and residual thrombosis 2 weeks after interventional therapy.

### Definitions

The severity of thrombosis was evaluated by venographic scoring systems from Society of Vascular Surgery (SVS scoring) that has been used in prior DVT studies [18]. Involvement of the following deep venous segments should be specified as follows: inferior vena cava, common iliac vein, external iliac vein, common femoral vein, femoral vein and popliteal vein. The patency of each segment was assessed depending on the results of the Venography. Assign grade: 0 = patent; 1 = subsegmental, non-occlusive thrombus; 2 = subsegmental, occlusive thrombus; 3 = occlusive thrombus throughout the length of the segment.

Technical success was defined as complete coverage of the thrombosis lesion by CDT or PMT and restored patent deep venous blood flow. Immediate clinical improvements referred to a decrease in pain or swelling of the affected extremity within 24 h of intervention. The differences in limb circumferences between DVT limb and the contralateral limb at 10 cm above the superior margin of patella and 15 cm below the inferior margin of patella were measured before and after the operation to judge the clinical effect. The degree of thrombus removal was graded by calculating the percentage reduction in patient total thrombus score and classified as grade I, II and III (see Table 2) [18]. Grade II/III was defined as substantial thrombus reduction. Hemorrhage was recorded as complications, divided into mild hemorrhage including wound hemorrhage or visible hematoma and severe hemorrhage including intracranial hemorrhage or gastrointestinal hemorrhage.

**Table 2:** Procedural characteristics and clinical outcomes in hospital

Variable	CDT (*n* = 65)	PMT (*n* = 49)	PMT + CDT (*n* = 35)	*χ*^*2*^*/F/Kruskal–Wallis H*	*P* value	*P* (CDT vs PMT)	*P* (CDT vs PMT + CDT)	*P* (PMT vs PMT + CDT)
Length of hospital stays	6.7 ± 1.7	4.3 ± 2.0	4.9 ± 1.6	28.081	< 0.001	< 0.001	< 0.001	0.105
Stents	40(61.5%)	31(63.3%)	23(65.7%)	0.171	0.918			
RIVCF placement	35(53.8%)	25(51.0%)	20(57.1%)	0.309	0.857			
Total urokinase doses(U)	1460.0[1420.0,1520.0]	235.0[185.5,265.0]	660[600.0,720.0]	83.144	< 0.001	< 0.001	< 0.001	< 0.001
Total heparin doses(mg)	250[220.0,280.0]	89.0[76.0,99.0]	158.0[135.0,174.0]	81.439	< 0.001	< 0.001	< 0.001	< 0.001
Procedural time(min)	53.5 ± 5.4	88.3 ± 8.1	89.7 ± 7.2	499.436	< 0.001	< 0.001	< 0.001	0.377
Thrombolysis time(h)	76.3 ± 14.7	0.18 ± 0.03	20.0 ± 4.6	893.337	< 0.001	< 0.001	< 0.001	< 0.001
Fatal hemorrhage^a^	3(4.6%)	2(4.1%)	1(2.9%)	0.183	0.913			
Thrombus removal grade^b^				2.712	0.607			
Grade III	49(75.4%)	36(73.5%)	30(85.7%)					
Grade II	11(16.9%)	7(14.3%)	3(8.6%)					
Grade I	5(7.7%)	6(12.2%)	2(5.7%)					

*CDT* catheter-directed thrombolysis

*PMT* percutaneous mechanical thrombectomy

*RIVCF*: retrievable inferior vena cava filter

^a^Fatal hemorrhage including intracranial hemorrhage, gastrointestinal bleeding

^b^The degree of thrombus removal was graded by calculating the percentage reduction in patient total thrombus score (ie, the difference between the baseline and post-procedural thrombus scores divided by the baseline score). Thrombus removal grade was classified as follows: 1) minimal or no thrombolysis as grade I: < 50% thrombus removal and reduction in thrombus score, partial thrombolysis as grade II: 50%–99% thrombus removal and reduction in thrombus score, and complete thrombolysis as grade III: 95–100% thrombus removal and reduction in thrombus score

### Follow-up

Clinical follow-up were assessed by ultrasound on the 1st, 3rd, 6th, and 12th month after the procedure and annually thereafter. The primary endpoints were recurrence of DVT, and severity level of PTS during the follow-up. The secondary endpoints were in-hospital (or 30-day) mortality, all-cause mortality, other major complications in the hospital (30-day) and during the follow-up, including intracranial hemorrhage, gastrointestinal hemorrhage and symptomatic pulmonary embolisms, stent occlusion or restenosis.

Initial patency was defined as patent deep venous blood flow confirmed by ultrasound and restenosis of deep vein or stent less than 50%. Additional imaging (venography or computed tomography venography) was performed for clinically suspected recurrence. Clinical symptoms and signs during the follow up were statistically analyzed by Villalta score [19], including 5 symptoms –pain, spasm, limb heaviness, paresthesia, skin itching and 6 signs –pretibial area edema, subcutaneous sclerosis, pigmentation, gastrocnemius tenderness, new varicose veins, skin redness and swelling. Each symptom and sign was scored as 0, 1, 2, 3 according to the severity level as non-occurrence, mild, moderate and severe. The total score ≥ 5 points is diagnosed as PTS, 5–14 points are defined as mild to moderate PTS, and ≥ 15 points or the presence of venous ulcer is defined as severe PTS.

### Statistical analysis

Continuous data was expressed as mean ± standard error. Data was evaluated with regard to normal distribution (Kolmogorov–Smirnov Test) and homogeneity of variances (Levene's Test). For analysis of differences between the groups, One-Way ANOVA was used, and a significant one-way ANOVA result will be followed up with post-hoc tests (SNK-q test). In the case of inhomogeneity of variances or deviation from the normal distribution, Kruskal–Wallis ANOVA on Ranks was used. Data was analyzed using SPSS V 13.0 (SPSS, Inc, Chicago, USA), and a two-tailed *P* value < 0.05 was considered statistically significant.

## Results

### Clinical characteristics

A total of 149 patients (male 97) were enrolled and assigned to PMT (first applied) + CDT (35/149, 24.5%), CDT alone (65/149, 43.6%) and PMT alone (49/149, 32.9%) groups. The average age was 62.7 ± 12.3 years old. The proportion of sub-acute DVT in PMT + CDT group was significantly higher than that in CDT group and PMT group (χ^*2*^ = 7.889, *p* = 0.019, PMT + CDT vs CDT *p* = 0.032, PMT + CDT vs PMT *p* = 0.005). The number of days from having symptoms to admission in the three groups was 15.0 ± 7.7 (CDT), 15.6 ± 7.5 (PMT) and 19.1 ± 5.9 (PMT + CDT). Numerically, there were slightly higher proportion of patients with longer duration of symptoms in PMT + CDT group compared to the other two groups (F = 3.710, *p* = 0.027, PMT + CDT vs CDT *p* = 0.009, PMT + CDT vs PMT *p* = 0.033). The proportion of May-Thurner Syndrome in the PMT group was lower than that in the other two groups (χ^*2*^ = 9.048, *p* = 0.011, PMT vs CDT *p* = 0.026, PMT + CDT vs PMT *p* = 0.005). The SVS Scoring was 6.98 ± 3.24 in CDT group, 7.29 ± 3.39 in PMT group and 9.91 ± 2.83 in PMT + CDT group. The SVS Scoring was significantly higher in PMT + CDT group than that in the other two groups (F = 10.386, *p* < 0.001, PMT + CDT vs CDT *p* < 0.001, PMT + CDT vs PMT *p* < 0.001) (Table 3).

**Table 3:** Patient characteristics

Characteristics	CDT (*n* = 65)	PMT (*n* = 49)	PMT + CDT (*n* = 35)	*χ*^*2*^/*F*	*P*	*P* (CDT vs PMT)	*P* (CDT vs PMT + CDT)	*P* (PMT vs PMT + CDT)
Gender male	40(61.5%)	34(69.4%)	23(65.7%)	0.765	0.682			
Age, years	62.6 ± 13.6	61.69 ± 11.7	64.0 ± 10.6	0.364	0.695			
Days from symptoms to admission	15.0 ± 7.7	15.6 ± 7.5	19.1 ± 5.9	3.710	0.027	0.680	0.009	0.033
Duration of symptoms				7.889	0.019	0.372	0.032	0.005
Acute(≤ 14d)	29	26	8					
Sub-acute(> 14d to ≤ 28d)	36	23	27					
Involved left side	55(84.6%)	38(77.6%)	28(80.0%)	0.957	0.620			
Leg pain	50(76.9%)	36(73.5%)	28(80.0%)	0.495	0.781			
Leg swelling	61(93.8%)	45(91.8%)	34(97.1%)	1.015	0.602			
Combined with PE	5(7.7%)	3(6.1%)	1(2.9%)	0.938	0.626			
May-Thurner Syndrome	44(67.3%)	23(47.1%)	27(77.1%)	9.048	0.011	0.026	0.321	0.005
SVS Scoring^a^	6.98 ± 3.24	7.29 ± 3.39	9.91 ± 2.83	10.386	< 0.001	0.620	< 0.001	< 0.001

Continuous data are presented as means ± standard deviation; Categorical data are given as counts (percentage)

*CDT* catheter-directed thrombolysis, *PMT* percutaneous mechanical thrombectomy, *PE* pulmonary embolism

^a^*SVS*: society of vascular surgery

### Procedural characteristics

The study shows a longer time of hospital stay in patients who received CDT only (6.7 ± 1.7 days) as compared with PMT + CDT patients (4.9 ± 1.6 days, *p* < 0.001) and PMT patients (4.3 ± 2.0 days, *p* < 0.001) (F = 28.081, *p* < 0.001). The procedural time was 88.3 ± 8.1 min in PMT group and 89.7 ± 7.2 min in PMT + CDT group, with no statistically significant difference (*P* = 0.377); however, it was longer than that in CDT group (53.5 ± 5.4 min), with statistical significance (both *P* < 0.001). The thrombolysis time was 76.3 ± 14.7 h in CDT group, 0.18 ± 0.03 h in PMT group and 20.0 ± 4.6 h in PMT + CDT group, with statistically significant differences among the three groups (*P* < 0.001 respectively). The use of stents in the three groups had no difference (χ^*2*^ = 0.171, *P* = 0.918). The total urokinase dose and heparin dose continued to be higher in patients that underwent PMT + CDT when compared with PMT patients (*P* < 0.001 respectively), but lower when compared with CDT patients (F = 83.144 for urokinase dose, *p* < 0.001, F = 81.439 for heparin dose, *P* < 0.001). Protective RIVCF was used in 35(53.8%) CDT patients, 25(51.0%) PMT patients and 20(57.1%) PMT + CDT patients to avoid further thrombus embolization during the procedure. The stents were used in 40(61.5%) CDT patients, 31(63.3%) PMT patients and 23(65.7%) PMT + CDT patients. There was no significance of RIVCF and stents placement among the three groups (Table 2).

### Clinical outcomes

All the patients had technical success and immediate clinical improvements. The degree of thrombus removal is shown in Table 2. The mean follow-up was 34.9 ± 9.1 months (range, 19–53 months) for the three groups. DVT recurrence/stent thrombosis happened on 14 (21.5%) patients in CDT group, 4 (8.2%) patients in PMT group and 2 (5.7%) patients in PMT + CDT group. The proportion of DVT recurrence/stent thrombosis in CDT group was significantly higher compared to PMT + CDT group (*P* = 0.04), but failed to reach the significant level compared to PMT group (*P* = 0.053). A total of 91.4% (60/65) of patients in CDT group, 87.8% (43/49) in PMT group, and 94.3% (33/35) in PMT + CDT group showed substantial thrombus reduction rate (grades II/III). There were no statistical differences in the thrombus removal grades between three groups (χ^*2*^ = 2.712, *P* = 0.607). The severity of PTS was the highest in CDT group (χ^*2*^ = 14.459, *P* = 0.006) compared to PMT group (*P* = 0.029) and PMT + CDT group (*P* = 0.006). The severity of PTS did not differ significantly between PMT group and PMT + CDT group (*p* = 0.428). The details are shown in Table 4.

**Table 4:** Outcomes during the follow up

Variable	CDT (*n* = 65)	PMT (*n* = 49)	PMT + CDT (*n* = 35)	*χ*^*2*^*/F/Kruskal–Wallis H*	*P* value	*P* (CDT vs PMT)	*P* (CDT vs PMT + CDT)	*P* (PMT vs PMT + CDT)
Follow-up(month)	36.4 ± 9.1	35.4 ± 9.0	31.1 ± 8.2					
PTS				14.459	0.006	0.029	0.006	0.428
0–4	43(66.2%)	43(87.8%)	32(91.4%)					
5–14	14(21.5%)	4(8.2%)	3(8.6%)					
≥ 15	8(12.3%)	2(4.1%)	0					
DVT recurrence/stent thrombosis	14(21.5%)	4(8.2%)	2(5.7%)	6.640	0.036	0.053	0.04	0.667

*CDT* catheter-directed thrombolysis

*PMT* percutaneous mechanical thrombectomy

*PTS* post-thrombotic syndrome

*DVT* deep venous thrombosis

Only 1 patient died of lung carcinoma 16 months after the treatment in CDT group. A total of 6 bleeding complications occurred after the operations, 3 patients after CDT (two with wound hemorrhage and one with gastrointestinal hemorrhage), 2 patients after PMT (one with wound hemorrhage and one with visible hematoma) and 1 patient after PMT + CDT (with wound hemorrhage). All the hemorrhage patients were successfully treated. There was no in-hospital occurrence of death, symptomatic pulmonary embolisms, or other severe procedure-related hemorrhage.

The differences in limb circumferences between DVT limb and the contralateral limb before and on the 1st and 3rd day after the operation are listed on supplement Table 2. Preoperatively, there was no significance regarding the differences in limb circumferences in three groups (F = 0.289, *P* = 0.749). The postoperative differences in limb circumferences decreased in all three groups compared to the preoperative differences. There was no difference among the three groups in limb circumferences above or below the knee (F = 1.008, *P* = 0.367).

## Discussion

Due to different treatments, a significant proportion of DVT patients will have venous insufficiency. Compared with conventional anticoagulation therapy, thrombolytic therapy can rapidly reduce thrombus burden [4], potentially preserve venous vascular function and reduce the risk of PTS [20]. Improvement of endovascular techniques makes it possible to use more approaches to increase success rate on DVT patients. In this retrospective study in our single center, the endovascular treatment of CDT, PMT and PMT + CDT was involved to provide clinicians with more evidence and technical details. In this study, patients in PMT + CDT group were featured with longer time from symptoms to admission, higher SVS scoring, but lower DVT recurrence/stent thrombosis rate and lower PTS severity, which provides data support and educates clinicians on the application of combined PMT + CDT therapy.

### Deficiency of CDT and PMT alone in DVT treatment

Patients that have been treated with CDT often have good venous function and health conditions [21]. However, there are a number of studies demonstrating that CDT also have high risk of recurrence and complications such as fatal hemorrhage and more severe PTS [22]. In this study, patients in the CDT group had longer length of hospital stays and higher dosage of anticoagulation and thrombolytic drugs, and showed the greatest number of DVT recurrence/stent thrombosis and severe PTS cases among the three groups. The possible explanation was that the CDT technique itself could not recover the blood flow immediately and provide timely protection for venous valve function. This deficiency might lead to the recurrence of thrombosis.

Compared with CDT, PMT is an efficient technology that directly removes thrombus to reduce the clot burden [23]. Because of shorter treatment time, lower lytic agent doses and less risk of hemorrhage, PMT has become more and more attractive. The therapeutic efficacy assessment of AngioJet, which is featured with rheolytic thrombectomy system—a major technique in PMT, is needed.

AngioJet has been used in our center since year 2013 as one of PMT systems. The proposed thrombectomy time of AngioJet was 600 s (10 min), however, it was found that many residual thrombus remained in deep venous after PMT; when DVT was involved in a wide range and high venographic scores, the thrombectomy time would exceed 10 min, and the treatment required to be carried on with CDT therapy after PMT. In our study, 23% (35/149) DVT patients had such condition and accepted PMT + CDT treatment accordingly.

### Possible indication of PMT + CDT

An increasing number of comparative studies has focused on PMT and CDT [24]. However, it is not very clear whether the indications of PMT and CDT are exactly the same and what kind of patients with DVT are suitable for each therapy. With the worldwide application of AngioJet, the indication of combined application of PMT + CDT need clarification. Liu G, et al. reported that the combination of PMT and CDT has higher thrombolysis efficacy and lower risk of PTS than using AngioJet alone [25]. In this study, the patients in PMT + CDT group got more days from symptoms to admission and sub-acute patients took up higher percentage compared with the other two groups. The patients in PMT + CDT group also got higher SVS scoring. These results indicate that the patients in PMT + CDT group have more severe conditions and have higher treatment difficulty. The May-Thurner Syndrome rate in PMT group was much lower than the other two groups, which means that PMT alone may not be suitable for the treatment of DVT combined with May-Thurner Syndrome. These patients were transferred to PMT + CDT group instead. In our retrospective study, combination of PMT + CDT was compared with PMT or CDT alone to provide necessary clinical data for the standardized use of PMT and/or CDT. According to the results, the possible indication of PMT + CDT could be concluded as follows: 1) Sub-acute DVT; 2) High SVS scoring; 3) DVT combined with May-Thurner Syndrome.

### Better clinical outcomes of PMT + CDT compared with each of them alone

Compared with CDT, PMT was a new method of endovascular therapy. The comparative results of CDT and PMT varied in different studies. CDT can strongly reduce the incidence of PTS and improve overall survival for patients with DVT [8]. PMT has been shown to lower both thrombolytic drug dosage and hemorrhagic risks. Few studies focus on the effect of PMT + CDT, and they concluded that combination of PMT and CDT has higher thrombolysis efficacy and lower the risk of PTS at 1 year [10]. In our study, though the patients in PMT + CDT group got longer duration of symptoms and higher SVS scoring, the combination of PMT + CDT could reduce the severity of PTS and the rate of DVT recurrence. However, PMT + CDT can’t reduce the application of stents and RIVCF. The thrombus removal grade and clinic signs were not significantly different from that in the other two groups. These results showed that PMT + CDT may improve the short-term outcomes but only demonstrate non-inferiority comparing with the other two groups. During the follow up, the mid-long term outcomes of PMT + CDT in protecting patients from PTS and thrombosis recurrence were significantly better than using PMT or CDT alone.

### Limitation

Our study is a retrospective study instead of a randomized controlled study, which means that the current grouping cannot completely rule out selection offsets and the verification of positive conclusions requires a prospective randomized controlled trial in the future. In our study, the combination of PMT + CDT was performed in one order, i.e. PMT followed by CDT, and the reverse order was not tested in this study, as results, the effect of PMT after CDT treatment was not clear.

## Conclusions

This investigation shows that the therapeutic effect of different interventional treatments on DVT patients is still controversial. PMT combined with CDT may benefit DVT patients with sub-acute DVT, high SVS scoring and combined May-Thurner Syndrome in our center.

### Supplementary Information

Supplementary Material 1. 

## Data Availability

The datasets used and/or analysed during the current study are available from the corresponding author on reasonable request.
