# Supplementary material for: Which one is the best in treating deep venous thrombosis —— percutaneous mechanical thrombectomy, catheter-directed thrombolysis or combination of them?
Source: J Cardiothorac Surg. 2024 Jul 5;19:423. doi: 10.1186/s13019-024-02908-3 (PMC11225378; doi:10.1186/s13019-024-02908-3)
Supplement: Supplementary file 1 — Supplementary Material 1. [file 13019_2024_2908_MOESM1_ESM.pdf]

Changhai Hospital review and ethics Committee

Certification

The clinical multi-center research project applied by Professor Qing-sheng Lu of Vascular Surgery in our hospital: the indications and effect evaluation of filter placement after deep vein thrombosis is planned to be implemented from January 1, 2018 to December 31, 2018.

After review, the research follows the principles of fairness and justice strictly, fully embodies the rights and interests of subjects, and ensures that the research will not put subjects at unreasonable risks. The entire study will not involve animal experiments. The research is in compliance with our country's current policies and regulations concerning human or animal medical ethics research, and is now approved for application.

Hereby certify.

Changhai Hospital review and ethics Committee

November 16, 2017

业务专用章

168 Chang Hai road, Yangpu District, Shanghai, China
Tel&Fax: 86-21-31162338, postcode: 200433, Email: changhaiec@126.com
